# Characteristics of Esophageal Cancer Cases in Tanzania

**DOI:** 10.1200/JGO.2016.006619

**Published:** 2017-04-27

**Authors:** Elia J. Mmbaga, Katrina V. Deardorff, Beatrice Mushi, William Mgisha, Megan Merritt, Robert A. Hiatt, Julius Mwaiselage, Li Zhang, Katherine Van Loon

**Affiliations:** **Elia J. Mmbaga**, **Beatrice Mushi**, and **William Mgisha**, Muhimbili University of Health and Allied Sciences; **Julius Mwaiselage**, Ocean Road Cancer Institute, Dar es Salaam, Tanzania; **Katrina V. Deardorff**, **Megan Merritt**, **Robert A. Hiatt**, **Li Zhang**, and **Katherine Van Loon**, University of California, San Francisco, San Francisco, CA.

## Abstract

**Purpose:**

Age-standardized incidence rates for esophageal cancer (EC) in East Africa have been reported as disproportionately high compared with the worldwide incidence of nine per 100,000 population. This study aimed to characterize EC cases seen at Muhimbili National Hospital and Ocean Road Cancer Institute in Dar es Salaam, Tanzania.

**Methods:**

Demographic, clinical, and treatment variables were abstracted from charts of patients who received care for a diagnosis of EC at one or both institutions between 2011 and 2013. Categorical data were summarized as frequency counts and percentages. Continuous data were presented as medians and ranges. To compare men and women, Pearson’s χ^2^ and two-sample *t* tests were applied.

**Results:**

Seven hundred thirty-eight unique cases of EC were identified, of whom 68% were men and the median age was 60 years (range, 19 to 95 years). Notably, 93 cases (13%) were ≤ 40 years old at diagnosis. Squamous cell carcinoma was the dominant histology, comprising 90% of cases with documented histopathology. However, 34% of cases with a diagnosis of EC were not pathologically confirmed. The stage was documented as locoregional in 4% of cases, locally advanced in 20% of cases, metastatic in 14% of cases, and unknown in 63% of cases. Of 430 patients who received treatment at Ocean Road Cancer Institute, 76% were treated with radiation, 44% were treated with chemotherapy, 3% underwent a cancer-related surgical procedure, and 10% of cases received no cancer-directed therapy. The median overall survival for all patients was 6.9 months (95% CI, 5.0 to 12.8), regardless of stage at presentation.

**Conclusion:**

Between 2011 and 2013, cases of EC represented a large clinical burden at both institutions.

## INTRODUCTION

Although the reported worldwide age-standardized incidence rate for esophageal cancer (EC) is nine cases per 100,000 population per year,^1^ this statistic does not reflect remarkable geographic variations in incidence rates. Currently, > 80% of cases and deaths from EC occur within developing countries. One of the most striking features of EC is the presence of defined high-incidence geographic regions, including locales in northern China, northeastern Iran, eastern South America, and South Africa.^2,3^ Eastern Africa was recently described as another high-incidence geographic area for EC,^4^ with incidence and mortality rates significantly higher than in western, middle, or northern Africa.^5^

Cancer is not a reportable disease in Tanzania, with limited data available on burden. In an era in which increasing attention and resources are being directed toward noncommunicable diseases in low- and middle-income countries, data regarding the burden of EC to the health care system are needed to begin building the capacity to provide care for this deadly disease. With a population of approximately 4.4 million persons, Dar es Salaam is the most highly and densely populated urban area in Tanzania and has the highest average population growth rate since 2002 (5.6%).^5^ To better understand the clinical characteristics of EC in Tanzania and the associated care burden, this study aimed to characterize the EC cases seen at two major referral hospitals in Dar es Salaam, Tanzania.

## METHODS

### Study Design

We conducted a retrospective chart review of all patients diagnosed with EC at Muhimbili National Hospital (MNH) and Ocean Road Cancer Institute (ORCI) in Dar es Salaam Tanzania between 2011 and 2013. Within Dar es Salaam, MNH is the public teaching hospital affiliated with Muhimbili University of Health and Allied Sciences and is a national referral hospital with 1,500 beds, admitting 1,000 to 1,200 inpatients per day and providing care to > 1,000 outpatients per week.^6^ Cancer cases warranting chemotherapy or radiation therapy are typically referred, following diagnosis, to ORCI, which is the only specialized facility for cancer treatment where radiation therapy is available in Tanzania.

### Study Population

All patients ≥ 18 years old who received care at either MNH or ORCI between 2011 and 2013 for a diagnosis of EC were included in this analysis. Cases of EC were identified at MNH using its computerized database. Cases of EC at ORCI were identified by reviews of the admission registry, chemotherapy logbook, and radiation therapy logbook. Because not all patients with a suspected diagnosis of EC undergo diagnostic biopsies for pathologic confirmation of malignancy as a result of the associated out-of-pocket costs, expanded criteria for inclusion were necessary. Cases were included on the basis of a histologically confirmed diagnosis of EC or a clinical diagnosis on the basis of barium swallow or an esophagogastroduodenoscopy without confirmatory biopsy.

### Data Collection

This retrospective chart review was approved by institutional review boards at University of California, San Francisco and Muhimbili University of Health and Allied Sciences. Available paper medical records for all patients with a documented diagnosis of EC were retrieved, and each was reviewed to confirm a documented diagnosis of EC. Cases without a clinical or pathologically documented diagnosis of EC were excluded. Names, ages, and medical record numbers of cases abstracted at MNH and ORCI were cross-checked to avoid double-counting patients who received care at both institutions. Each case was de-identified with a unique study identification number. Data were entered into Research Electronic Data Capture, a secure Web-based application for data storage.

Demographic, clinical, and treatment variables were abstracted from the medical records. Demographic variables included age at diagnosis, sex, ethnicity, and location of primary residence. Distance traveled from primary residence to Dar es Salaam was calculated by inputting the district of origin into Distance Calculator.^7^ Zones of Tanzania were delineated according to categories used by the 2010 Tanzania Demographic and Health Survey. Primary referral hospitals were categorized as private, regional, or district.

Clinical variables included symptoms at presentation, anatomic location of primary tumor, histologic subtype, and disease stage. Date of diagnosis was recorded as date of the first confirmatory test result. If dates for diagnostic tests were not found, the date of first presentation for care related to the EC diagnosis was used. In cases in which the anatomic location of the tumor in the proximal, middle, or distal esophagus was not documented, the documented distance from incisors was converted to anatomic location: tumors with a proximal border at < 18 cm were classified in the upper esophagus; 18 to < 32 cm were classified in the middle esophagus; and ≤ 32 cm were classified in the lower esophagus. For those cases who received treatment for the diagnosis of EC, details on surgical procedures, administration of radiation and/or chemotherapy, or receipt of palliative care were abstracted.

### Statistical Methods

Demographic data and clinical characteristics were summarized with descriptive statistics. Categorical data were summarized as frequency counts and percentages, and continuous data were presented as medians and ranges. To compare between men and women, Pearson’s χ^2^ and two-sample *t* tests were applied for categorical data and continuous data, respectively. Overall survival (OS) was assessed by the Kaplan-Meier method, and the comparisons of OS among the different groups of subjects were done by log-rank test. Statistical significance was declared at *P* < .05. All analyses were performed using the statistical computing software R (http://www.r-project.org).

## RESULTS

### Demographic Characteristics

From hospital records at MNH and ORCI, 738 unique cases of EC from Tanzania were identified as having received care at one or both institutions between 2011 and 2013. Nearly all cases were documented as African (n = 686; 93%), and 68% (n = 468) of cases were men. The overall median age at diagnosis was 60 years (range, 19 to 95 years). Notably, 93 cases (13%) were ≤ 40 years old at diagnosis.

Demographic characteristics of all EC cases, according to institution, are summarized in Table 1. More than half of all cases (n = 427, 59%) reported having a primary residence in the Coastal Zone of Tanzania. The median distance traveled from primary residence to MNH was 194 km (range, 3 to 1,416 km) and 455 km (range, 4 to 1,416 km) from primary residence to ORCI.

**Table 1:**
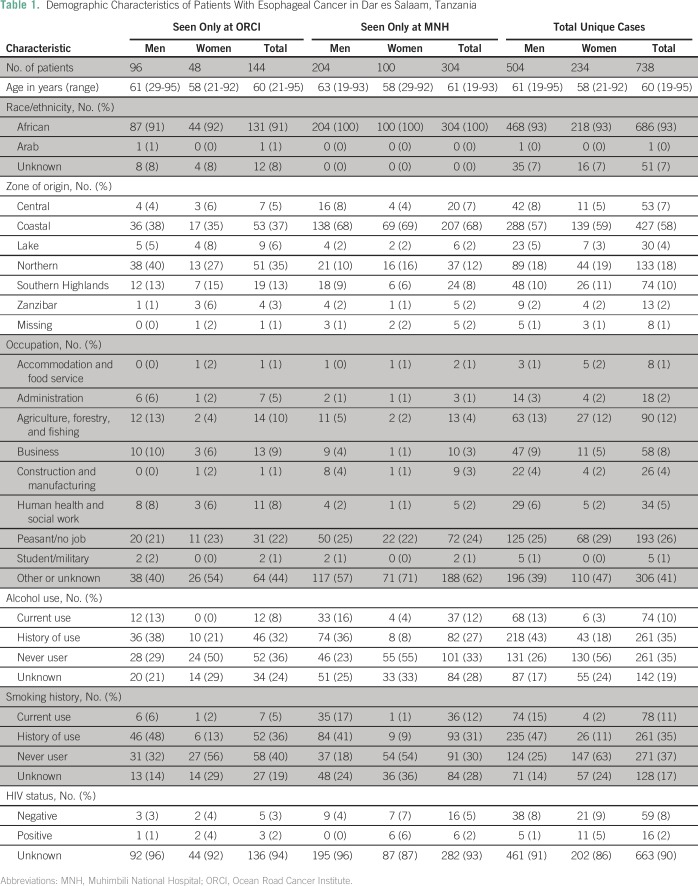
Demographic Characteristics of Patients With Esophageal Cancer in Dar es Salaam, Tanzania

Among individuals for whom smoking status was documented (433 men and 177 women), 29% (n = 124) of men versus 83% (n = 177) of women were documented as never smokers (*P* < .001). Among individuals for whom alcohol consumption history was documented (417 men and 179 women), 31% (n = 131) of men versus 73% (n = 130) of women were documented as having no previous or current alcohol consumption (*P* < .001). Reporting of patient occupation was identified in 59% (n = 432) of medical records. Among all EC cases, 26% (n = 193) of EC cases were documented as being small-scale subsistence farmers or unemployed (eg, peasants), and an additional 12% (n = 90) were documented as working in agriculture, forestry, or fishing industries.

HIV serostatus was documented for only 10% (n = 75) of all cases. Only 2% (n = 16) of all EC cases were documented as HIV positive. Among patients with documented HIV status, 21% (16 of 75) were HIV positive.

### Clinical Characteristics

Of 594 cases seen first at MNH, 266 (45%) were referred from regional hospitals, 205 (35%) from district hospitals, and 53 (9%) from private hospitals. For 144 cases referred directly to ORCI for treatment without being seen at MNH, 56 (39%) were referred from private hospitals and 44 (30.6%) from regional hospitals.

Table 2 summarizes the clinical characteristics of EC cases. Squamous cell carcinoma (SCC) was the dominant histology, comprising 90% of cases with documented histopathology. However, 34% of cases with a documented diagnosis of EC were on the basis of clinical information only and were not pathologically confirmed. Diagnosis of EC was made by endoscopy with biopsy for 79% and 88% of cases presenting to MNH and ORCI, respectively. Anatomic location of the tumor was documented in the proximal or middle esophagus in the majority of cases. Dysphagia to solids was documented in 100% of cases.

**Table 2:**
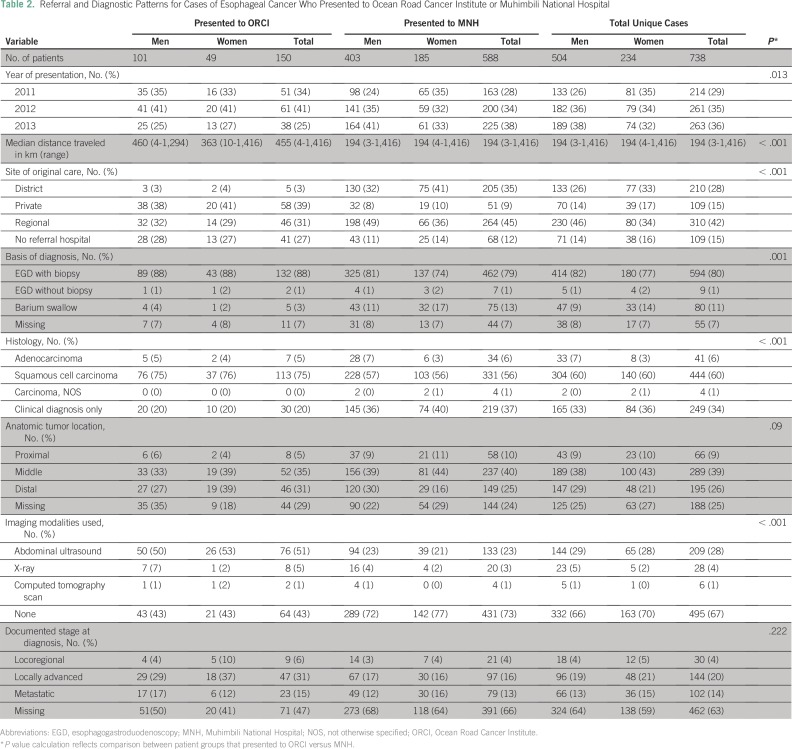
Referral and Diagnostic Patterns for Cases of Esophageal Cancer Who Presented to Ocean Road Cancer Institute or Muhimbili National Hospital

Abdominal ultrasound was the most common imaging modality used for staging and was performed in 51% of cases at ORCI and 23% of cases who presented to MNH. The majority of cases (67%) underwent no staging imaging. Cross-sectional imaging (eg, computed tomography scans) was performed only in 1% of all cases. Stage was documented as locoregional in 4% of cases, locally advanced in 20% of cases, metastatic in 14% of cases, and unknown or missing in 63% of cases. Of all cases with documented stage seen at ORCI, 37% were documented as localized or locoregional disease versus 20% at MNH.

### Treatment Characteristics

**Fig 1:**
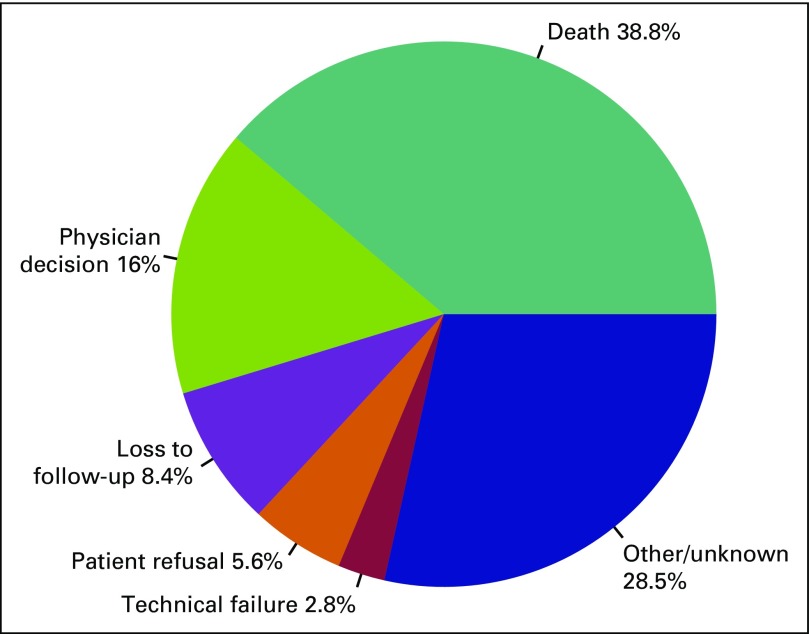
Documented reasons for early discontinuation of treatment.

Among the 430 cases who received treatment for their diagnosis of EC at ORCI, intent of treatment was documented as curative in 46% (n = 196) of cases, palliative in 46% (n = 199) of cases, and undocumented in 8% (n = 35) of cases. Of all cases at ORCI, 76% were treated with radiation therapy (n = 326), and 44% of cases received chemotherapy (n = 190), with either palliative or curative intent. Forty-two percent of patients (n = 181) received both chemotherapy and radiation as part of their treatment. Ten percent of cases (n = 39) received no cancer-directed therapy and were treated with palliative care only. Only 3% of patients (n = 13) underwent a cancer-related surgical procedure. Of the 430 cases treated at ORCI, 214 (50%) did not complete the initially recommended treatment. Reasons for discontinuation of treatment are summarized in Figure 1.

### Overall Survival

**Fig 2:**
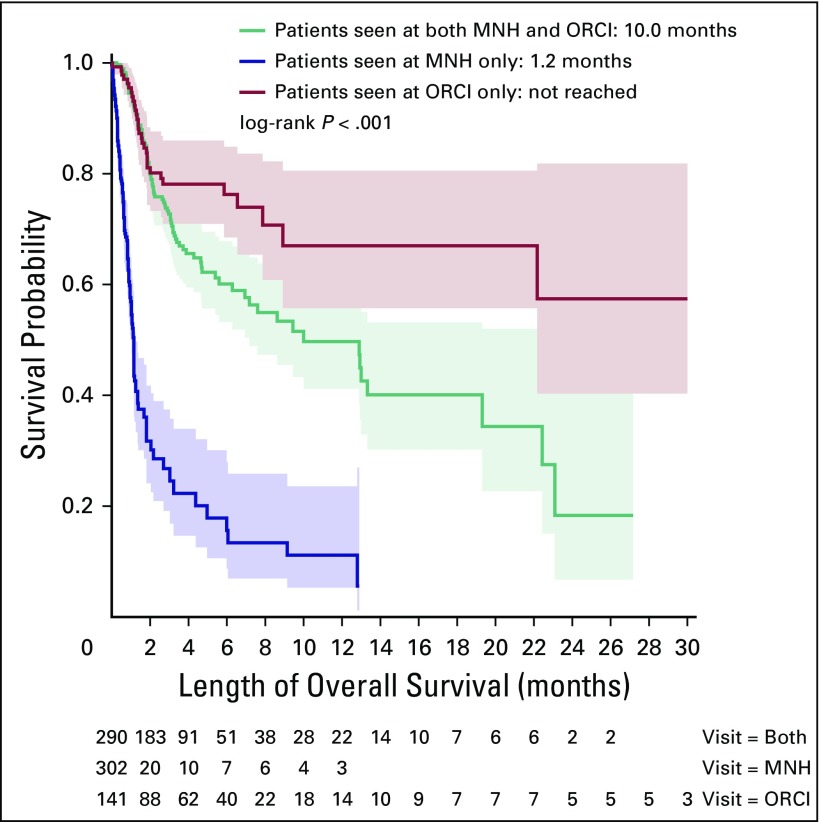
Overall survival according to site(s) where patients received care. MNH, Muhimbili National Hospital; ORCI, Ocean Road Cancer Institute.

The median follow-up was 1.3 months (range, 0 to 35.8 months) across all sites; however, duration of follow-up differed according to site (0.5 months at MNH *v* 3.3 months at ORCI *v* 2.8 months for patients receiving care at both institutions). Of the 304 cases who presented to MNH and did not receive treatment at ORCI, 40% were reported as dead at date of last contact. Of the 434 cases who received care at ORCI, 30% were reported as dead at date of last contact. Kaplan-Meier curves for OS are shown in Figure 2. The median OS for all patients was 6.9 months (95% CI, 5.0 to 12.8), regardless of stage at presentation. The median OS for cases who received care only at MNH was 1.1 months (95% CI, 1.0 to 1.2) versus 10 months (95% CI, 7.2 to 22.4) for cases who presented to both institutions (*P* < .001). The median OS could not be calculated for those who presented only to ORCI because 50% of patients did not reach the primary end point during follow-up.

## DISCUSSION

Among the 738 patients with EC who received care at two major referral hospitals in Dar es Salaam, Tanzania between 2011 and 2013, 68% were men, and nearly all were of African descent. Of those with histologic confirmation, SCC was the dominant histology. Our findings regarding the characteristics of patients with EC in Tanzania are consistent with previous reports from this region.^8^ Presentation with advanced symptoms was common, comparable to reports from other parts of Eastern Africa.^9^ This is probably in part as a result of barriers to cancer treatment that exist in Tanzania, including prohibitive costs of diagnostic tests and challenges in accessing care.

Although we cannot infer causal associations from these data, it is notable that documented rates of current or previous alcohol consumption, smoking, and HIV infection among EC cases are higher than in the Tanzania population at large.^10,11^ Although HIV status was documented for only a small proportion of patients, 21% of those with documented HIV testing were reported as HIV positive. This is substantially higher than the overall approximately 5% seroprevalence of HIV in Tanzania among adults ages 15 to 49 years and merits further evaluation, particularly in light of recent data from a case-control study in Zambia that reported HIV infection as a risk factor for EC.^12^ Additionally, the Coastal and Northern zones of Tanzania lead in referrals to both institutions. These regions are in closest proximity to Dar es Salaam; thus, referral bias probably accounts for this finding. The Kilimanjaro region (within the Northern Zone) of Tanzania is, however, an area of active investigation as a high-incidence area within Tanzania.^13,14^ Further inquiry into the role of tobacco and alcohol exposures, HIV infection, as well as unique geographic exposures as possible contributors to the high incidence of EC in Tanzania is warranted.

One of the most striking findings is the proportion of unusually young cases, with 13% of cases ≤ 40 years old at the time of diagnosis. Among the patients ≤ 40 years old, 56% were men, and 95% of cases with a histopathologic diagnosis were SCC. Although many patients were ≤ 40 years old, a younger population age structure in African countries may significantly contribute to a younger age at cancer diagnosis, compared with other settings. However, the unusually high representation of young individuals could also point to a possible contribution from genetic factors and/or environmental exposures to the high incidence of this disease. No significant demographic differences among the patients ≤ 40 years old at diagnosis were identified, when compared with those older than 40 years.

A significant difference in OS was identified between cases who received care only at MNH versus those who received care at ORCI. This difference in outcomes between patients who established care at ORCI is probably in part as the result of patient selection. Notably, 37% of patients who presented to ORCI were documented as having locoregional or locally advanced disease, versus 20% of those who presented to MNH, suggesting that patients with metastatic disease are less likely to be referred to ORCI. Moreover, the sickest patients probably died at MNH shortly after presentation and before referral. Although we acknowledge the limitations of this comparison as a result of lack of detailed information regarding prognostic factors, including stage and performance status, and nonstandardization of staging procedures and treatment decisions across providers, differences in survival suggest that patients referred to ORCI for consideration for treatment are appropriately selected in this resource-constrained setting. Given that death is a major reason for loss to follow-up in Africa, we acknowledge that OS may be overestimated for patients for whom vital status was unknown at the time of censoring.

Among patients who received cancer-directed treatments at ORCI, a vast majority were treated with radiation, either with or without concurrent chemotherapy. Intent of treatment was documented by providers as curative in half of cases, often despite unknown stage; this perhaps reflects provider optimism in the absence of staging capabilities. Whereas systemic chemotherapy would be the mainstay of clinical care for advanced EC in a developed country, low use of chemotherapy in this cohort may reflect accessibility of radiotherapy compared with chemotherapy drugs. Similarly, self-expanding metal stents have been demonstrated as feasible and effective in palliating inoperable EC in other African settings.^15^ Currently, stents are not widely available in Tanzania for palliation of this disease, as the result of prohibitively high commercial pricing and import tax rates, which are further compounded by the socioeconomic status of patients.

Despite patient selection for referral to ORCI, nearly half of patients treated at ORCI did not complete recommended therapies. The high frequency of treatment cessation as the result of deterioration of the patient’s clinical status suggests that, despite careful selection, many patients were in fact not well enough to tolerate available treatments or that additional supportive-care resources are needed to safely perform these treatments. Moreover, this finding highlights the need to evaluate the role for radiation therapy in the management of EC, particularly in patients with advanced disease. We infer from these data that radiation therapy is used in the majority of cases, even those with advanced or metastatic disease, as palliation for obstruction. However, successful administration of this type of therapy typically requires a multidisciplinary support team, including nursing, nutrition, and social work services. Further inquiry into whether this is the safest and most efficacious approach in a resource-constrained setting, in which appropriate supportive care may not be readily available, is planned. Specifically, the feasibility and cost-effectiveness of palliative stenting as an alternative to radiation to relieve obstruction merits further inquiry in this and similar settings.

Several limitations of this study must be acknowledged. Because of the retrospective nature, data on multiple variables of interest were limited by the absence of documentation in the medical records; therefore, our findings were potentially subject to reporting bias. Specifically, 34% of patients did not have any documented pathologic diagnosis in the medical record. A majority of clinical diagnoses were accompanied by results from a barium swallow as a surrogate for histopathologic confirmation; however, the possibility that noncancer cases were erroneously included exists. Similarly, disease stage was not documented for 63% of cases, reflecting that cross-sectional imaging is not readily accessible or routinely used as part of clinical evaluation for EC. With only 1% of EC cases undergoing cross-sectional imaging, we did not conduct analyses stratified by stage because of the high likelihood of inaccuracies.

Data abstraction at both institutions was limited to only medical records that were retrievable, and cases for which a chart was not located were omitted. A reported number of 573 and 581 cases of EC were seen at ORCI in 2012 and 2013, respectively (unpublished data); thus, it is probable that available charts underrepresent all cases of EC. Additionally, follow-up data for many patients were incomplete, highlighting the need for improved patient-tracking systems to reduce loss to follow-up and to make possible further inquiry to identify barriers to care for patients with EC, such as stigma, distance to access medical care, or costs of care. Finally, we acknowledge that although this study included EC cases from a national referral hospital and the only specialized cancer treatment center in Tanzania, this sampling probably underrepresents all EC cases in Tanzania, and it is not possible to derive any conclusions about incidence rates for the population at large.

In conclusion, during 3 consecutive years, a large number of patients with EC were seen at MNH and/or ORCI in Dar es Salaam, Tanzania. Patients traveled long distances to seek care and often presented with advanced disease. Acknowledging limitations as a result of the retrospective nature of this study, these findings will provide initial data to allow for a review of the diagnostic and therapeutic strategies applied in this resource-constrained setting. With increased awareness and education regarding diagnosis and treatments of EC in this high-incidence area, this knowledge will enable improved allocation of resources for capacity building, diagnosis, and treatment. In addition, our future research will evaluate the etiology of the high incidence of EC in Tanzania, with particular inquiry into its occurrence among younger adults.
